# The Institutional Worker

**Published:** 1911-11-04

**Authors:** 


THE
### INSTITUTIONAL WORKER
**Being a Special Supplement to "The Hospital"**

### Editor's Notices.

**Contributions:**

Contributions should be written, or preferably typed, on one side of the paper only, and all articles sent in are accepted upon the distinct understanding that they are forwarded to THE HOSPITAL only.

The Editor cannot undertake to return MSS. not used, but when a stamped directed envelope is enclosed the MSS. may be returned if a special request is made.

Accepted articles and paragraphs of news will be paid for after publication at the scale rate.

**Address:**

To prevent delay all contributions and letters on editorial business must be addressed exclusively to the Editor, "The Hospital," 29 Southampton Street, Strand, London, W.C. It is important that this regulation shall be strictly observed.

**Correspondence:**

Correspondence on all subjects is invited. The name and address of correspondents must be given as a guarantee of good faith, but not necessarily for publication.

**Special Articles:**

Special articles are invited, and questions, inquiries, and paragraphs upon all matters relating to the work, administration, and management of general, special, mental, fever, cottage and convalescent hospitals, sanatoria, homes, institutions, societies and organisations for the treatment or care of the sick, injured and dependents of all classes. Every matter affecting the interests and welfare of the staff of all grades working in these institutions will receive special consideration.

**Administrative Medicine, Research and Items of News:**

Special terms will be paid for approved articles on subjects in this wide field by experts who have made some section of it their special study and interest. We wish to give prominence to every movement tending to advance accuracy of diagnosis, efficiency in treatment, and the development of modern methods to eradicate disease and promote the welfare of the suffering.

**A Bureau of Information:**

We invite inquiries and applications for information and help in providing for that numerous class of sufferers who require special care or house-room which they cannot obtain in their own homes or secure for themselves. **We cannot, however, prescribe or recommend practitioners.**

**Books for Review:**

Publishers are particularly requested to send advance proofs of any new books of importance whenever possible, as the Editor has made arrangements to publish immediate reviews on a new plan.

**Photographs, Plans, Blocks and Illustrations:**

It is requested that wherever possible MSS. may be accompanied by illustrations in any of the above forms. The name of the sender and of the article to which it belongs should be written on the back of each photograph, drawing, or block for purposes of identification.

**Local Papers:**

Newspapers containing reports or news paragraphs should be marked and addressed to the Sub-Editor.

**The Coupon System:**

A coupon will be found at the bottom of the third inside page of the cover each week. This coupon must be attached to every question or inquiry to which an answer is desired in THE HOSPITAL.

### Manager's Notices.

Letters relating to the publication, sale, and advertising departments of THE HOSPITAL must be addressed to the Manager, "The Hospital," The Hospital Building, 28 and 29 Southampton Street, Strand, London, W.C.

**Circulation and Sale:**

THE HOSPITAL is on sale at all newsagents and bookstalls every Friday, price one penny. The rates of subscriptions, post free from THE HOSPITAL office, have been reduced and are now as follows:—

| | United Kingdom s. d. | The Colonies and Abroad s. d. |
|---|---|---|
| For one year ... ... ... | 6 6 | 8 8 |
| ,, six months ... ... | 4 0 | 5 0 |
| ,, three months ... ... | 2 0 | 2 6 |

**Subscriptions,** which may begin at any time, are payable in advance. Cheques and Post-Office orders, crossed London County and Westminster Bank, Covent Garden branch, should be made payable to the Manager of THE HOSPITAL and addressed to him at The Hospital Building, 28 and 29 Southampton Street, Strand, London, W.C.

**Advertisements:**

All advertisements for THE HOSPITAL must reach the Manager not later than Wednesday morning in each week. For scale of charges see pages v and 2.

**Classified Advertisements:**

All small advertisements will be classified, for this section of the paper is widely read and of special interest. We wish to encourage those wanting appointments who are attracted to institutional work, and therefore offer them the reduced rate of *twenty-one words and under for* 1s., and *for every ten and part of ten words beyond,* 6d.

### Points for Institution Authorities.

THE economical management of an Institution depends very greatly upon purchasing supplies in the cheapest market.

IT is often impossible locally to secure the highest quality at the lowest prices.

IN consequence, a section will be reserved in this part of the paper for announcements of Institutions inviting Tenders for Contracts.

THIS will enable Authorities of Institutions to secure tenders for supplies from all over the country, and ensure purchases at the lowest possible prices consistent with quality.

THE charge for Tenders is 6s. per inch, and for this small outlay many pounds may be saved on supplies of all descriptions.

**Special Rates** will be quoted for those institutions which agree to send all their vacancy, contract, and public notices to THE HOSPITAL each year, so as to make it a file of such announcements for ready reference.

### Points for Trade Advertisers.

THE HOSPITAL is the organ of Institutions, whether of a Voluntary Character or those under Local Government, Poor Law and Municipal Authorities.

IT is read by all Executive Officials and those responsible for the Construction, Management, and Equipment of Public Establishments.

IT is also the Journal of the worker engaged in the Medical, Health and Preventive services.

THERE is no medium more appropriate and effective in the promotion of business in these directions than THE HOSPITAL.

IT is the Authoritative Journal of Administrative Medicine and of Institutional Life, and is relied upon by all connected with or interested in the Prevention of Disease, the alleviation of the sick, and the general health and well being of the community.

### APPOINTMENTS VACANT.

### Poor-Law Vacancies.

### Appointments Vacant.

ASSISTANT ATTENDANTS (£20), West Ham Union. Mr. T. Smith, C. to G., Leytonstone, N.E. Nov. 9.

ASSISTANT MATRON (£20), Goole Union. Mr. R. Haldenby, Clerk to the Children's Home Committee, Bank Chambers, Goole. Nov. 7.

ASSISTANT NURSE (£22), Cricklade and Wootton Bassett Union. Mr. H. Bevir, C. to G., Wootton Bassett. Nov. 10.

ASSISTANT NURSE (£25), Exeter Guardians. Mr. A. Snell, C. to G., Exeter. Nov. 8.

ASSISTANT NURSES (£21), Banbury Union. Mr. E. L. Fisher, C. to G., Banbury. Nov. 7.

ASSISTANT NURSE (£26), Knaresborough Union. Mr. J. Smith, C. to G., Knaresborough. Nov. 6.

ASSISTANT NURSERY ATTENDANT (£18), Lewisham Union. Mr. H. C. Mott, C. to G., 394 High Street, Lewisham. Nov. 11.

CHARGE ATTENDANT (£30), West Ham Union. Mr. T. Smith, C. to G., Leytonstone, N.E. Nov. 9.

CHARGE NURSE (£28), Rothwell Isolation Hospital. Mr. W. B. Pindar, Leek Street, Hunslet. Nov. 4.

CHARGE NURSE (£24), City of London Guardians. Apply Matron, Workhouse, 42 Clifden Road, N.E.

CHILDREN'S ATTENDANT (£17), Farnham Union. Miss Tisdell, Superintendent Nurse, The Infirmary, Farnham.

FEMALE ATTENDANT (£18), Bakewell Union. Mr. A. Hawes, C. to G., Bakewell. Nov. 4.

FEMALE IMBECILE ATTENDANT (£25), Lincoln Union. Mr. W. B. Danby, C. to G., Lincoln. Nov. 7.

FOSTER-MOTHERS (£26), West Ham Union. Mr. T. Smith, C. to G., Leytonstone, N.E. Nov. 9.

HEAD NURSE (£32), Southwark Union. S. Wood, C. to G., Union Offices, Ufford Street, Blackfriars, S.E.

HEAD NURSE (£35), Glanford Brigg Union. Mr. F. C. Hett, C. to G., Brigg. Nov. 8.

INFIRM WOMEN'S ATTENDANT (£25), North Bierley Union. Mr. W. G. Cooper, C. to G., 4 Town Hall Street, Bradford. Nov. 7.

JUNIOR ASSISTANT CLERK (£45), Shoreditch Guardians. Mr. R. Clay, C. to G., 213 Kingsland Road, N.E. Nov. 6.

LABOUR MASTER (£30), Uxbridge Union. Mr. C. Woodbridge, C. to G., Uxbridge. Nov. 10.

LABOUR MISTRESS (£25), Southwark Union. Mr. S. Wood, C. to G., Ufford Street, Blackfriars, S.E. Nov. 7.

MASSAGE SISTER (£36), Birmingham Infirmary, Dudley Road. Apply Matron. Nov. 13.

MASTER AND MATRON (£50 and £35), Fareham Union. Mr. A. Laker, C. to G., Fareham. Nov. 10.

MASTER AND MATRON (£60 and £40), Glanford Brigg Union. Mr. F. C. Hett, C. to G., Brigg. Nov. 8.

MATRON'S ASSISTANT (£16), Bromyard Union. Mr. F. W. Nicholl, C. to G., Bromyard. Nov. 6.

MOTHERS' AND INFANTS' ATTENDANT (£25), Southwark Union. Mr. S. Wood, C. to G., Ufford Street, Blackfriars, S.E. Nov. 7.

NURSE (£30), Wigan Union. Mr. H. G. Ackerley, C. to G., Wigan. Nov. 9.

NURSE (£30), Pewsey Union. Mr. R. Dixon, C. to G., Pewsey. Nov. 4.

NURSE (£35), Sleaford Union. Mr. E. Clements, C. to G., Sleaford. Nov. 4.

NURSE (£25), Worksop Union. J. Snow Whall, C. to G., 66 Bridge Street, Worksop. Nov. 7.

NURSES (£30), West Derby Union. H. P. Cleaver, C. to G., Brougham Terrace, Liverpool. Nov. 9.

SISTER (£35), Wandsworth Union. F. W. Piper, C. to G., Union Offices, St. John's Hill, Wandsworth.

SUPERINTENDENT NURSE (£45), Isle of Thanet Union. Mr. C. Taylor, C. to G., Minster, near Ramsgate. Nov. 9.

SUPERINTENDENT OF VAGRANTS (£29), Durham Union. Mr. H. E. Ferrens, C. to G., Durham. Nov. 4.

WARD SISTER (£30), Hammersmith Guardians. Clerk to Guardians, 206 Goldhawk Road, Shepherd's Bush.

### Situations Vacant.

ASSISTANT COOK (£20), Hammersmith Guardians. The C. to G., 206 Goldhawk Road, Shepherd's Bush, W. Nov. 13.

ASSISTANT ENGINEER (£45), Hammersmith Guardians. The C. to G., 206 Goldhawk Road, Shepherd's Bush, W. Nov. 13.

ASSISTANT PORTER AND LABOUR MASTER (£30), Hull Guardians. Mr. R. H. Winter, C. to G., Hull. Nov. 7.

COOK-ATTENDANT (£25), West Ham Union. Mr. T. Smith, C. to G., Leytonstone, N.E. Nov. 9.

COOK (£25), Bosmere and Claydon Union. Mr. R. M. Cook, C. to G., 6 Providence Street, Ipswich. Nov. 13.

DISTRICT GAS FOREMAN (state salary), Birmingham T.C. Fittings Superintendent, Gas Department, Birmingham. Nov. 8.

KITCHENMAID (£18), West Ham Union. Mr. T. Smith, C. to G., Leytonstone, N.E. Nov. 9.

LAUNDRESS (£30), Uxbridge Union. Mr. C. Woodbridge, C. to G., Uxbridge. Nov. 10.

NIGHT PORTER (28s. weekly), Holborn Union. Mr. J. H. Battersby, C. to G., Clerkenwell Road, E.C. Nov. 10.

PORTRESS (£21), Durham Union. Mr. H. E. Ferrens, C. to G., Durham. Nov. 4.

PORTER (£30), Guisborough Union. Mr. W. Richardson, C. to G., Guisborough. Nov. 7.

### Institutional and Administrative Appointments.

OPHTHALMIC SISTER (£30), King Edward VII.'s Hospital, Cardiff. Apply Matron. Nov. 18.

MATRON (£50), Fleetwood Cottage Hospital. Hon. Secretary, Milton Lodge, Fleetwood. Nov. 12.

### Miscellaneous Vacancies.

### Situations Vacant.

CLERK TO SCHOOL MANAGERS (£107), Glamorgan Education Committee. Mr. T. M. Franklen, Clerk to the County Council, Cardiff. Nov. 6.

NURSE (£30), Lanchester Joint Hospital Board. Mr. W. H. Ritson, Clerk to the Board, Lanchester. Nov. 6.

NURSE FOR PHYSICALLY DEFECTIVE CHILDREN (£80), L.C.C. Education Offices, Victoria Embankment, W.C. Nov. 4.

SCHOOL NURSE (£75), Tottenham Education Committee. W. Mallinson, Clerk, Education Offices, S. Tottenham. Nov. 11.

SCHOOL NURSE (£80), Borough of Gillingham (Kent) Education Committee. A. Johns, secretary, 4 Gardiner Road, Gillingham. Nov. 10.

### Municipal Vacancies.

### Appointments Vacant.

ASSISTANT NURSE (£25), Newton Abbot Joint Isolation Hospital. Mr. J. Underhill, Clerk to the Committee, Newton Abbot. Nov. 7.

NURSE (£35), Brandon and Byshottles U.D. Council. A. A. Luxmoore, 5 North Bailey, Durham. Nov. 13.

PROBATIONER (£14), Lancaster Corporation. Matron, Sanatorium, Lancaster.

PROBATIONER (£10), Newton Abbott Joint Isolation Hospital. Mr. J. Underhill, Clerk to the Committee, Newton Abbot. Nov. 7.

SANITARY INSPECTOR (£150), Heaton Norris U.D.C. Mr. F. W. Brooke, Clerk to the Council, Heaton Moor, Lancs. Nov. 7.

SISTER (£33), Rothwell Isolation Hospital. W. B. Pindar, Leek Street, Hunslet, Leeds. Nov. 4.

### Situations Vacant.

WARDMAID (£20), Newhaven Urban Dist. Council. E. Knightley, Council's Office, Newhaven. Nov. 6.

